# Supplementary material for: Plasma p-tau212 antemortem diagnostic performance and prediction of autopsy verification of Alzheimer’s disease neuropathology
Source: Nat Commun. 2024 Mar 23;15:2615. doi: 10.1038/s41467-024-46876-7 (PMC10960791; doi:10.1038/s41467-024-46876-7)
Supplement: Supplementary file 1 — Supplementary Information [file 41467_2024_46876_MOESM1_ESM.pdf]

Supplementary Table 1. Peptides detected in the antibody specificity experiment by IP-MS. Only peptides with p-tau212 present were detected.

Extended-peptide standard[a]	Extended peptide sequence[b]	*m/z*	Charge	Detected peptide
170-195-pT181	RIPA(K)TPPAP(K)[T]PPSSGEPP(K)SGDRS	561.949	3+	
190-214-pS202	KSGDRSGYSSPG[S]PGTPGS(R)SRTPS	742.345	2+	
190-214-pT205	KSGDRSGYSSPGSPG[T]PGS(R)SRTPS	742.345	2+	
190-214-pS202+pT205	KSGDRSGYSSPG[S]PG[T]PGS(R)SRTPS	782.291	2+	
205-229-pT212	TPGSRSR[T]PSLPTPPT(R)EP(K)KVAVV	587.972	3+	X
205-229-pS214	TPGSRSRTP[S]LPTPPT(R)EP(K)KVAVV	587.972	3+	
205-229-pT217	TPGSRSRTPSLP[T]PPT(R)EP(K)KVAVV	587.972	3+	
205-229-pT212+pT217	TPGSRSR[T]PSLP[T]PPT(R)EP(K)KVAVV	614.627	3+	X
220-245-pT231	TREPKKVAVV(R)[T]PP(K)SPSSA(K)SRLQT	586.668	3+	

[a] Numbering according to tau 2N4R
[b] [X] amino acid with phosphate group, (X) isotope labelled amino acid; [$^{13}C_6$,$^{15}N_2$-Lys] or [$^{13}C_6$,$^{15}N_4$-Arg]; black letters are detected peptide after trypsinization, and red letters are extended sequence parts

Supplementary Table 2. Neurofibrillary tangles and neuropil threads count in slices of AD brains.

Tangles Count	p212	AT8		Tangles Count	p212	p217
**AD1**	17	17		**AD1**	6	6
**AD2**	10	10		**AD2**	7	7
**AD3**	39	36		**AD3**	18	18
**Threads Count**	**p212**	**AT8**		**Threads Count**	**p212**	**p217**
**AD1**	385	415		**AD1**	355	331
**AD2**	154	155		**AD2**	336	326
**AD3**	301	243		**AD3**	363	359

Supplementary Table 3. The %parallelism values for each subsequent dilution factor.

Plasma parallelism			
**Dilution/Sample**	**Sample 1**	**Sample 2**	**Sample 3**
2-fold	92.3	106.0	121.7
4-fold	78.6	84.0	118.7
**CSF parallelism**			
**Sample/%Parallelism**	**Sample 1**	**Sample 2**	**Sample 3**
32-fold	100.7	87.2	81.7
64-fold	97.4	87.5	91.0

Supplementary Table 4. CSF spike recovery - analytical validation of the accuracies of the p-tau212 assay to quantify signal from an exogenous source (spike) added to a primary sample.

CSF Recovery						
	**Analyte**	**Observed conc. (pg/ml)**		**Expected Concentration (pg/ml)**	**%CV conc.**	**% recovery**
		**Measured**	**Mean**			
**Sample 1**	Neat	10.857<br>10.943	10.9		0.5	
	+ high spike	35.324<br>40.416	37.87	39.14	9.5	96.7
	+ low spike	20.727<br>20.481	20.06	20.98	0.8	98.2
**Sample 2**	Neat	11.496<br>13.387	12.44		10.7	
	+ high spike	35.908<br>33.449	34.68	40.68	5	85.3
	+ low spike	21.332<br>25.087	23.21	22.52	11.4	103.1
**Buffer control**	Buffer + high spike	28.339<br>28.135	28.24		0.5	
	Buffer + low spike	9.801<br>10.351	10.08		3.9	

*Expected concentration was calculated as the sum of the sample and spike levels analyzed separately. The measured concentration is the response obtained for the true analyte concentration in the spiked sample.

#% recovery is the fraction of the measured to the expected concentration x 100%.

Supplementary Table 5. Plasma spike recovery - analytical validation of the accuracies of the p-tau212 assay to quantify signal from an exogenous source (spike) added to a primary sample.

Plasma Recovery							
	Treatment	Observed conc. (pg/ml)		Expected Concentration (pg/ml)	%CV conc.	% recovery
		Measured	Mean			
**Sample 1**	Neat	0.593<br>0.837	0.715		24.1	
	+ high spike	0.881<br>1.158	1.02	1.119	19.2	86.0
	+ low spike	0.859<br>0.892	0.875	0.997	2.7	82.9
**Buffer control for Sample 1**	Buffer + high spike	0.453<br>0.356	0.404		17.0	
	Buffer + low spike	0.311<br>0.254	0.282		14.5	
**Sample 2**	Neat	1.634<br>1.946	1.790		12.3	
	+ high spike	3.114<br>3.115	3.115	3.481	0.0	79.6
	+ low spike	2.473<br>2.334	2.404	2.511	4.1	94.0
**Buffer control for Sample 2**	Buffer + high spike	1.534<br>1.848	1.691		13.1	
	Buffer + low spike	0.623<br>0.820	0.721		19.3	

*Expected concentration was calculated as the sum of the sample and spike levels analyzed separately. The measured concentration is the response obtained for the true analyte concentration in the spiked sample.
#% recovery is the fraction of the measured to the expected concentration x 100%.

Supplementary Table 6 - Demographic characteristics of the BLSA-Neuropathology cohort

	**Controls**	**Asymptomatic**	**AD**
Sample size	12	15	20
Age, y	76.40 ± 15.47	84.50 ± 8.507	82.71 ± 9.873
Plasma – death interval	4.450 ± 2.767	2.871 ± 2.295	5.022 ± 3.464
Sex, F, *n (%)*	2/12 (16.7%)	5/15 (33.3%)	11/20 (55%)
Neuritic Plaques: Low/None	12/12 (100%)	0/15 (0%)	0/20 (0%)
Neuritic Plaques: Moderate	0/12 (0%)	3/15 (20%)	4/20 (25%)
Neuritic Plaques: High	0/12 (0%)	12/15 (80%)	16/20 (75%)
BRAAK 0-II	4/12 (33.3%)	1/15 (6.7%)	0/20 (0%)
BRAAK III-IV	8/12 (66.7%)	13/15 (86.7%)	6/20 (30%)
BRAAK V-VI	0/12 (0%)	1/15 (6.7%)	14/20 (70%)
Plasma p-tau181, pg/ml	7.7 (5.1 – 10.7)[c]	6.84 (4.3-13.8)	14.2 (9.4-18.4)[a]
Plasma p-tau231, pg/ml	10.8 (8.7-18.4)[c]	10.23 (8.1-19.2)	18.75 (11.8-23.9)[a]
Plasma p-tau212 (Simoa), pg/ml	1.2 (0.7-1.5)[c]	1.012 (0.8-2.1)[c]	4.022 (2.2-4.9)[a,b]

a – Different from Controls
b – Different from ASYMAD
c – Different from AD

Supplementary Table 7 - Demographic characteristics of the UCSD-Neuropathology cohort

	**Low Pathology**	**ADNC**	**Other Pathologies**	**ADNC + other**
Sample Size	8	21	19	19
Age at Death	81.6 ± 6.5	80.2 ± 9	77.7 ± 6.9	78.6 ± 8.5
CSF - Death interval	4.3 ± 2.1	4.6 ± 2.4	3.8 ± 1.7	4.8 ± 2.3
Sex F, *n*, %	4 (50%)	10 (48%)	8 (42%)	7 (37%)
Education (y)	12.8 ± 5.5	15.4 ± 4.3	17 ± 2.7	14.5 ± 2.8
Neuritic Plaques: Low/None	5 (62%)	0 (0%)	10 (53%)	0 (0%)
Neuritic Plaques: Moderate	2 (25%)	6 (29%)	8 (42%)	7 (37%)
Neuritic Plaques: High	1 (12%)	15 (71%)	1 (5%)	12 (63%)
Braak 0-II	4 (50%)	0 (0%)	5 (26%)	0 (0%)
Braak III-IV	4 (50%)	0 (0%)	9 (47%)	0 (0%)
Braak V-VI	0 (0%)	21 (100%)	0 (0%)	19 (100%)
LBD: Brainstem	0 (0%)	0 (0%)	1 (5%)	0 (0%)
LBD: Limbic	0 (0%)	0 (0%)	1 (5%)	2 (11%)
LBD: Neocortical	0 (0%)	0 (0%)	9 (47%)	13 (68%)
Hippocampal Sclerosis	0 (0%)	0 (0%)	1 (5%)	5 (26%)
FTLD	0 (0%)	0 (0%)	8 (42%)	0 (0%)
Other Pathology	0 (0%)	0 (0%)	0 (0%)	2 (11%)
CSF p-tau181	242.5 (178.3-480.3)[b]	906.5 (515.5-1012)[a,c]	316.7 (168.8-502.9)[b,d]	857.4 (547.8-2374)[c]
CSF p-tau231	373.7 (302.4-742.6)[b]	1332 (753.7-1883)[a,c]	469.9 (256.7-695.1)[b,d]	1644 (702.5-2165)[c]
CSF p-tau212	62.4 (46.8-169.2)[b,d]	444.6 (227.3-663.2)[a,c]	102.8 (40.8-169.0)[b,d]	441.2 (217-872.2)[a,c]
CSF p-tau217	2.2 (0.8-3.0)[b]	11.35 (8.7-18.3)[a,c]	3.97 (1.8-8.4)[b,d]	11.35 (6.2-18.8)[c]

a – Statistically different from Low pathology
b – Statistically different from ADNC
c – Statistically different from Other Pathology
d – Statistically different from ADNC + other pathology

Supplementary Table 8 - Demographic characteristics of the Gothenburg cohort

	**Aβ- controls**	**Aβ+ AD**
Sample size	14	16
Age, y	68.43 ± 9.835	76.63 ± 5.512*
Sex, F, n (%)	5/14 (35.7%)	9/16 (56.3%)
CSF Aβ42, pg/ml	1110 (672.8-1168)	457 (420.3-515)*
CSF total-tau, pg/ml	297 (175.8-354)	635 (496.5-999.3)*
CSF p-tau181 (Innotest), pg/ml	48 (32.5-57.3)	84 (71.8-100.5)*
Plasma p-tau212, pg/ml	1.0–(0.5 - 1.7)	3.8 (2.6-6.8)*
Plasma p-tau231, pg/ml	4.9 (4.4 - 6.6)	11.22 (9.1-16.1)*
Plasma p-tau181, pg/ml	4.488 (3.7 - 6.7)	8.377 (7.2-12.4)*

Supplementary Table 9 - Demographic characteristics of the Polish cohort

	**Aβ- controls**	**Aβ+ AD**
Sample size	21	74
Age, y	63.82 ± 7.72	69.86 ± 8.508*
Sex, F, *n (%)*	11/21 (52%)	52/74 (70%)
MMSE	25 (21-28.3)	18 (15-23.3)*
CSF Aβ40	16706 (12167-18040)	4965 (12693-19470)*
CSF Aβ42, pg/ml	1030 (833.5-1174)	233 (389.5-570.8)*
CSF Aβ42/Aβ40 ratio	0.065 (0.06-0.078)	0.033 (0.028-0.39)*
CSF total-tau, pg/ml	234 (2002.5-263.5)	565.5 (450-775.3)*
CSF p-tau181 (Innotest), pg/ml	46 (39-48.5)	95 (74.5-106.5)*
CSF p-tau212 (Simoa), pg/ml	38.55 (20.9-68.6)	384.5 (289.7-587.5)*
Plasma p-tau212, pg/ml	0.86 (0.43-2.2)	3.44 (2.1-5.6)*

Supplementary Table 10. Spearman correlations between plasma p-tau biomarkers and participants differentiated by diagnosis.

BLSA-Neuropathology cohort: diagnosis						
**Test - Plasma**	**Control versus AD**		**ASYMAD versus AD**		**Control versus ASYMAD**	
**Result/ analyte**	Fold change	P-value	Fold change	P-value	Fold change	P-value
**p-tau181**	2.15	0.0326	1.71	0.0771	1.26	0.8237
**p-tau231**	1.88	0.0215	1.52	0.0725	1.24	0.7423
**p-tau212**	3.92	<0.0001	3.19	<0.0001	1.22	0.9150

UCSD-Neuropathology cohort: diagnosis								
**Test - CSF**	**Low Path versus High ADNC**		**Low Path versus High ADNC+other**		**Other Path versus High ADNC**		**Other Path versus High ADNC+other**	
**Result/ analyte**	Fold change	P-value	Fold change	P-value	Fold change	P-value	Fold change	P-value
**p-tau181**	2.85	0.0178	2.51	0.0892	2.76	0.0018	2.43	0.0206
**p-tau231**	2.87	0.0299	2.79	0.0501	2.77	0.0034	2.71	0.0075
**p-tau212**	3.71	0.0062	3.50	0.0178	4.04	0.0002	3.82	0.0008
**p-tau217**	2.68	0.0163	2.41	0.0644	2.73	0.0005	2.46	0.0050

Supplementary Table 11. Spearman correlations between plasma p-tau biomarkers and AD burden in the brain.

UCSD-Neuropathology cohort									
**Biomarker**	**p-tau181**	**p-tau212**	**p-tau217**	**p-tau231**	**CSF Aβ42/40**	**CSF T-tau**	**Braak**	**CERAD**	**Thal**
**p-tau181**	x	0.98	0.95	0.98	-0.71	0.90	0.58	0.63	0.61
**p-tau212**	0.98	x	0.95	0.97	-0.68	0.88	0.67	0.67	0.63
**p-tau217**	0.95	0.95	X	0.94	-0.69	0.86	0.59	0.65	0.63
**p-tau231**	0.88	0.87	0.94	x	-0.74	0.89	0.59	0.62	0.61
**CSF Aβ42/40**	-0.71	-0.68	-0.69	-0.74	x	-0.58	0.47	-0.48	-0.59
**CSF T-tau**	0.90	0.88	0.86	0.89	-0.58	x	0.47	0.50	0.48
**Braak**	0.58	0.67	0.59	0.59	-0.47	0.47	x	0.69	0.60
**CERAD**	0.63	0.67	0.65	0.62	-0.48	0.50	0.69	x	0.67
**Thal**	0.61	0.63	0.63	0.61	-0.59	0.48	0.60	0.67	x

BLSA-Neuropathology cohort					
**Biomarker**	**p-tau181**	**p-tau212**	**p-tau231**	**CERAD**	**Braak**
**p-tau181**	x	0.62	0.83	0.32	0.40
**p-tau212**	0.62	x	0.69	0.39	0.46
**p-tau231**	0.83	0.69	x	0.34	0.46
**CERAD**	0.32	0.39	0.34	x	0.77
**Braak**	0.40	0.46	0.46	0.77	x

Supplementary Table 12. P-values for Spearman correlations between plasma p-tau biomarkers and AD burden in the brain.

UCSD-Neuropathology cohort									
**Biomarker**	**p-tau181**	**p-tau212**	**p-tau217**	**p-tau231**	**CSF Aβ42/40**	**CSF T-tau**	**Braak**	**CERAD**	**Thal**
**p-tau181**	x	2.9e-42	7.7e-32	1.4e-47	2.7e-11	7.1e-25	0.000001	1.7e-8	2.9e-7
**p-tau212**	2.9e-42	x	6.5e-32	3.1e-42	3.0e-10	2.4e-22	2.6e-9	1.1e-9	5.7e-8
**p-tau217**	7.7e-32	6.5e-32	X	3.9e-30	2.9e-10	2.0e-19	0.000001	7.9e-9	1.1e-7
**p-tau231**	1.4e-47	3.1e-42	3.9e-30	x	2.2e-12	1.6e-23	6.2e-7	3.0e-8	1.8e-7
**CSF Aβ42/40**	2.7e-11	3.0e-10	2.9e-10	2.2e-12	x	2.4e-7	0.000135	0.000036	5.3e-7
**CSF T-tau**	7.1e-25	2.4e-22	2.0e-19	1.6e-23	-2.4e-7	x	0.000134	0.000015	0.00008
**Braak**	0.000001	2.6e-9	0.000001	6.2e-7	0.000135	0.000134	x	1.4e-9	0.000001
**CERAD**	1.7e-8	1.1e-9	7.9e-9	3.0e-8	0.000036	0.000015	1.4e-9	x	2.2e-9
**Thal**	2.9e-7	5.7e-8	1.1e-7	1.8e-7	5.3e-7	0.00008	0.000001	2.2e-9	x
BLSA-Neuropathology cohort									
**Biomarker**	**p-tau181**	**p-tau212**	**p-tau231**	**CERAD**	**Braak**				
**p-tau181**	x	0.000003	5.1e-13	0.03	0.006				
**p-tau212**	0.000003	x	1.0e-9	0.007	0.001				
**p-tau231**	5.1e-13	1.0e-9	x	0.018	0.001				
**CERAD**	0.03	0.007	0.018	x	3.7e-10				
**Braak**	0.006	0.001	0.001	3.7e-10	x				

Supplementary Table 13. The coefficients of variation (%CV) Internal Quality Control samples.

Cohort	Internal Quality control (iQC) sample	% CV			
		**P-tau181**	**P-tau212**	**P-tau217**	**P-tau231**
Gothenburg	iQC 1	0.2%	3.2%	-	0.6%
	iQC 2	5.7%	3.6%	-	12.0%
Polish - CSF	iQC 1	-	3.3% within run and 7.1% between-run	-	-
	iQC 2	-	9.6% within run and 7.1% between-run	-	-
Polish - plasma	iQC 1	-	8.3% within run and 19.0% between-run	-	-
	iQC 2	-	19.0% within run and 19.9% between-run	-	-
BLSA-Neuropathology	iQC 1	8.1%	1.0%	-	1.3%
	iQC 2	4.6%	5.2%		3.8%
UCSD-Neuropathology	iQC 1	2.8%	5.7%	1.6%	1.1%
	iQC 2	2.7%	4.1%	14.7%	2.5%
Slovenia	iQC 1	-	13.7% within run and 13.7% between-run	4.5% within run and 17.8% between-run	-
	iQC 2	-	14.7% within run and 14.7% between-run	4.8% within run and 20.5% between-run	-

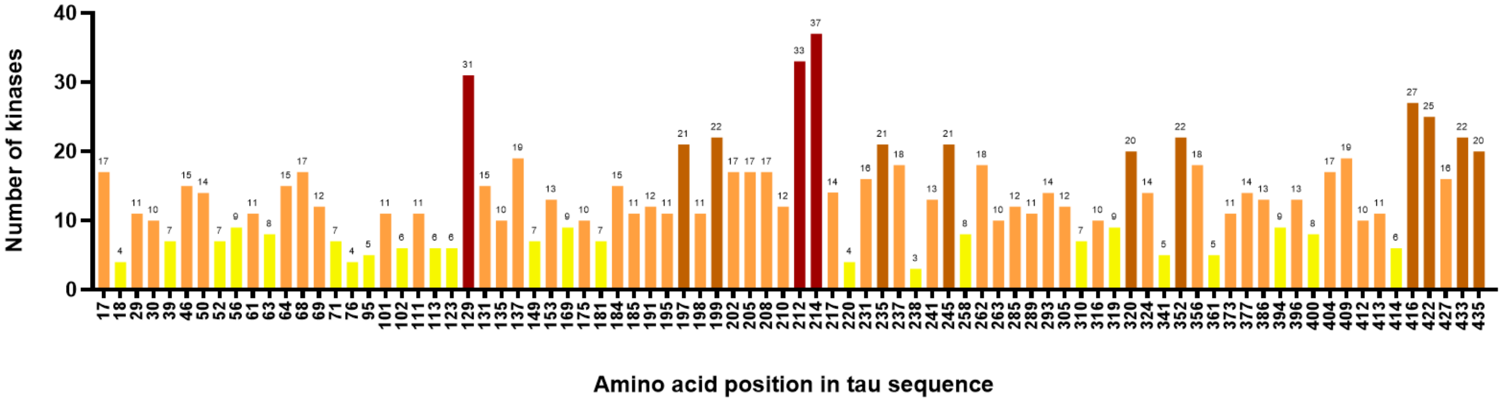

Supplementary Figure 1. Computational prediction of epitopes in full-length tau-441 protein phosphorylatable by unique kinases.
The figure shows the number of different kinases that are predicted to phosphorylate single epitopes in human tau protein 2N4R (Uniprot ID: P10636-8) created by the computational tool GPS 5.0. The epitopes are arranged in the order of amino acid positions in tau protein. The colour coding corresponds to the number of predicted kinases. The numbers at the top of each bar indicates the total number of predicted kinases. Bars are representing number of kinases that are predicted to phosphorylate single epitope. Source data are provided as a Source Data file.

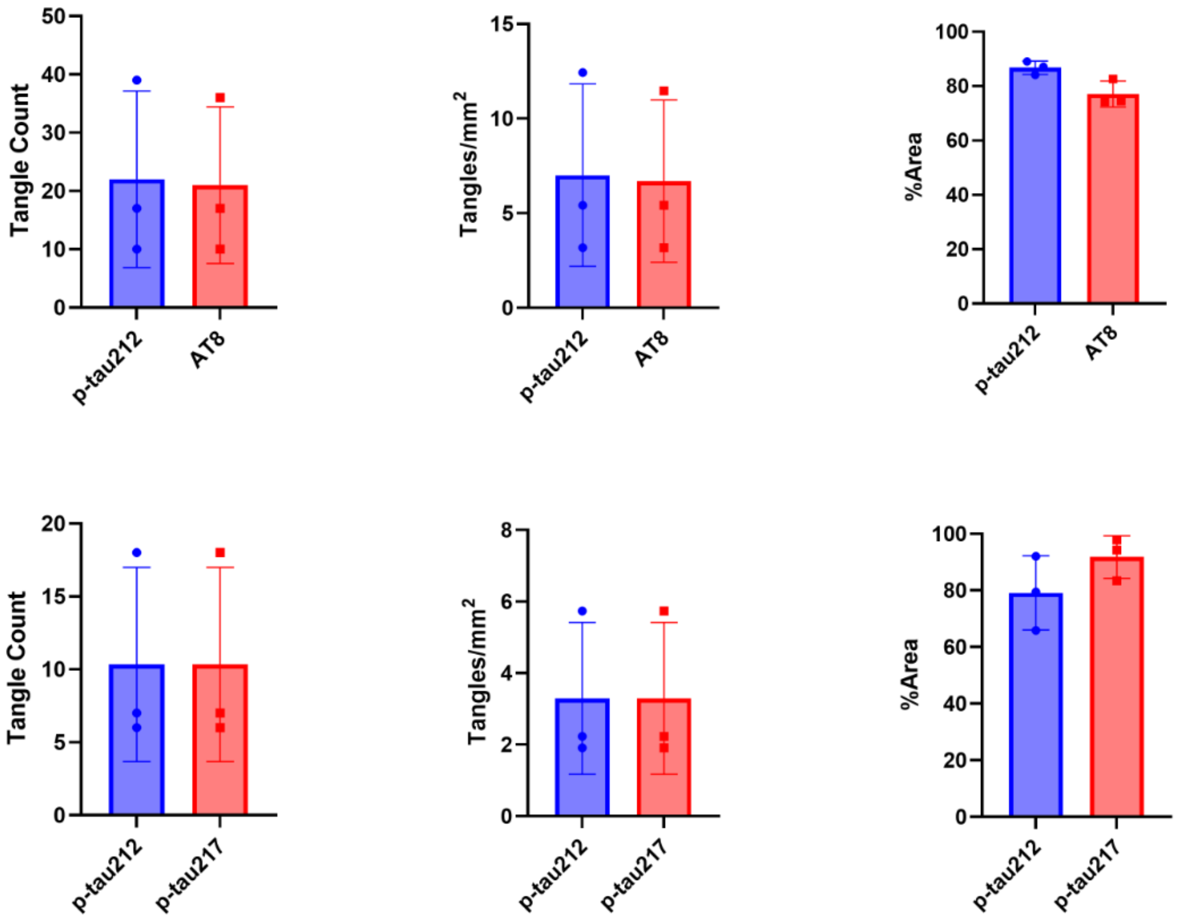

Supplementary Figure 2. Bar plot representation of tangle count and %area of tangles covered by each antibody in the immunofluorescent staining experiments.

Three independent AD brains were evaluated. In each plot, a single dot refers to data from one of these individuals. The data are presented as mean values, and the vertical bars show standard deviation. Source data are provided as a Source Data file.

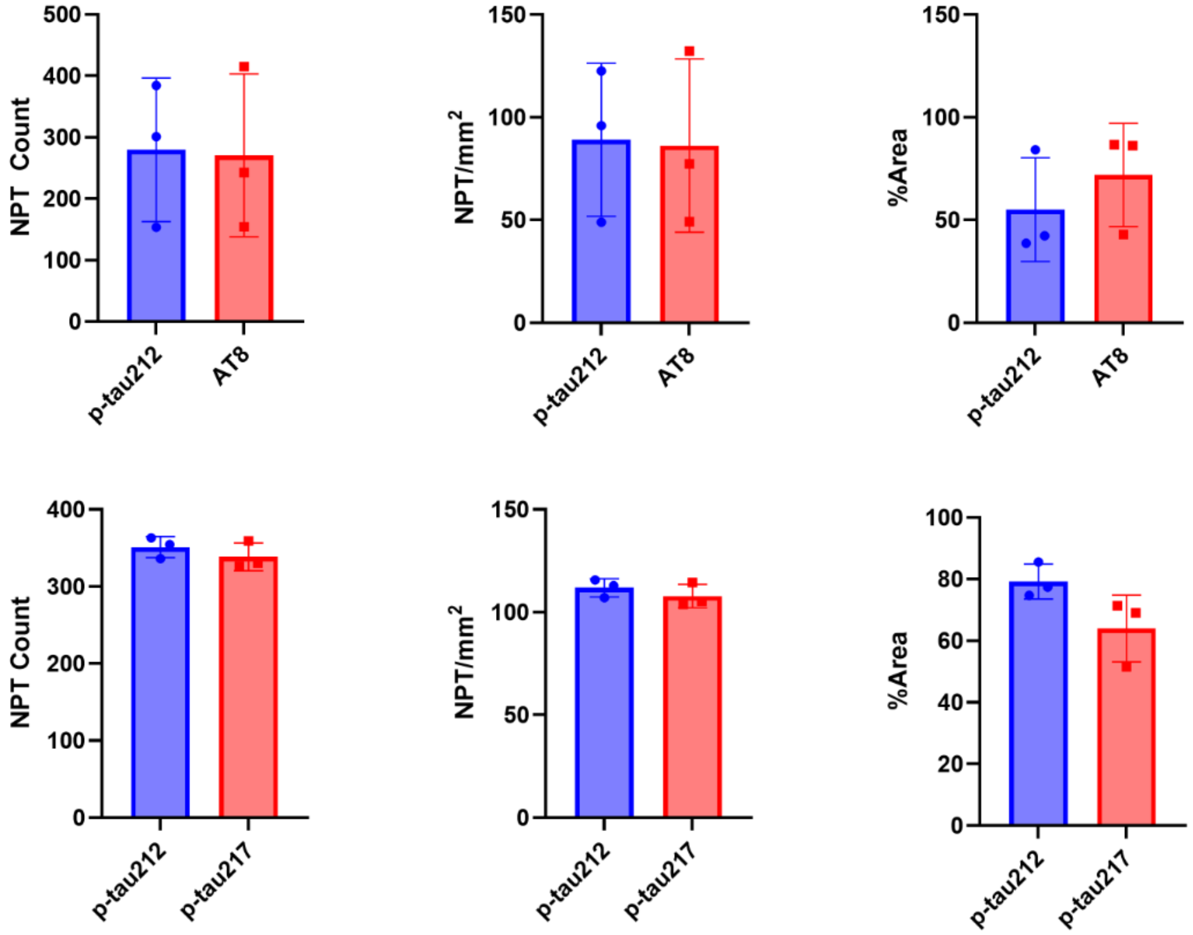

Supplementary Figure 3. Bar plot representation of neuropil threads count and %area of neuropil threads covered by each antibody in the immunofluorescent staining experiments.

Three independent AD brains were evaluated. In each plot, a single dot refers to data from one of these individuals. The data are presented as mean values, and the vertical bars show standard deviation. Source data are provided as a Source Data file.

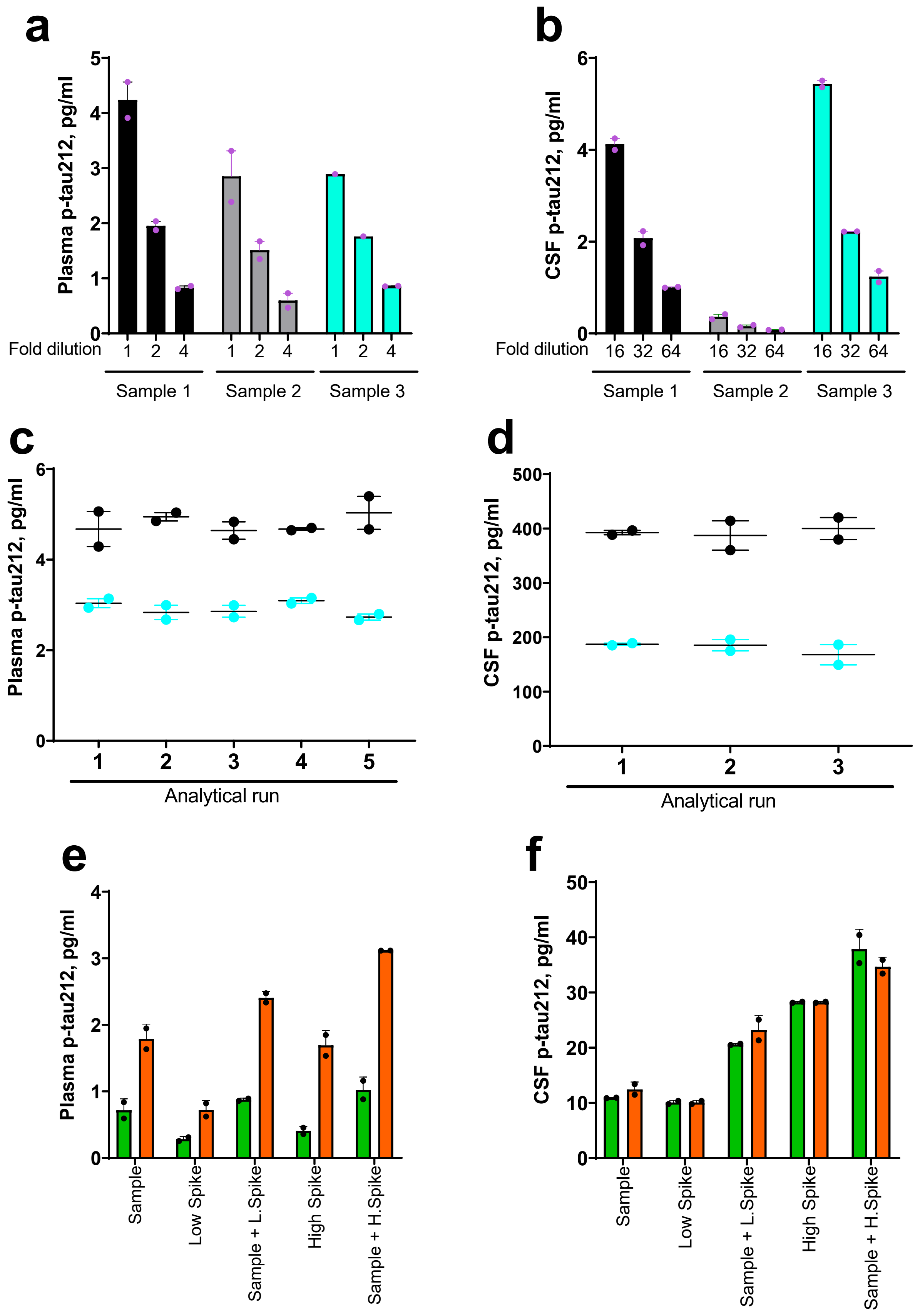
a
Plasma p-tau212, pg/ml
Fold dilution
Sample 1
Sample 2
Sample 3
b
CSF p-tau212, pg/ml
Fold dilution
Sample 1
Sample 2
Sample 3
c
Plasma p-tau212, pg/ml
Analytical run
d
CSF p-tau212, pg/ml
Analytical run
e
Plasma p-tau212, pg/ml
Sample
Low Spike
Sample + L.Spike
High Spike
Sample + H.Spike
f
CSF p-tau212, pg/ml
Sample
Low Spike
Sample + L.Spike
High Spike
Sample + H.Spike

Supplementary Figure 4. Technical validation of the plasma p-tau212 and p-tau217 assays developed in this study

a) and **(b)** Dilution linearity of the p-tau212 assay in plasma and CSF respectively. For each matrix, the plots show the measured concentrations in three unique samples with variable levels of the biomarker. Three equal-volume aliquots of each sample were prepared and either measured undiluted or diluted two- or four-fold (16-, 32- and 64-fold for CSF) with the assay diluent. Samples were run in duplicates, aside from plasma sample 3 which was in singlicates. The measured concentrations (without compensation for the fold dilution) are shown. %parallelism values for each subsequent dilution factor are presented in Supplementary Data Table 3. **(c)** and **(d)** Within- and between-run stability for plasma p-tau212. The plot shows the concentrations of two separate plasma samples were measured in duplicates in five independent analyses. For the sample with a mean concentration of 2.9 pg/ml, the within-run repeatability was 94.7% and the between-run repeatability was 93.6%. For the sample with mean concentration 4.8 pg/ml within-run and between run stability were both 92.4%. **(d)** Within- and between-run repeatability of the p-tau212 assay in CSF. The figure shows concentrations of aliquots of two independent CSF samples measured in duplicates in five separate analyses. For the sample with mean concentration 180.1 pg/ml within-run and between run stability were both 90.3%. For the sample with mean concentration 393.3 pg/ml within-run and between run stability were both 93.0%. Note that the concentration values shown in **(c)** and **(d)** have been adjusted for the dilution before sample measurement **(e)** and **(f)** Recovery of the p-tau212 assay in plasma and CSF respectively. For each matrix, the plot shows measured concentrations of the samples, concentrations of spiked CSF for plasma recovery or assay calibrator for CSF, and concentrations of samples with added low or high spikes. Data are presented as mean (SD). Source data are provided as a Source Data file.

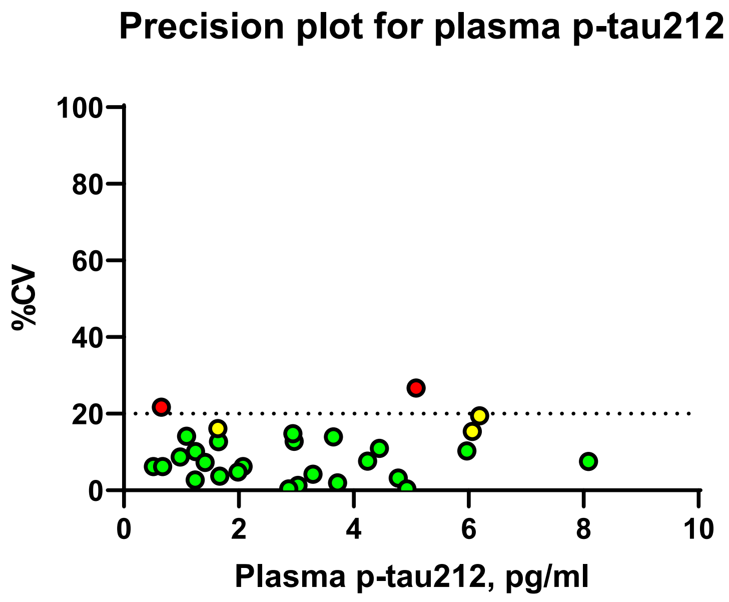

Supplementary Figure 5. Coefficients of variation for p-tau212 in de-identified plasma samples.

The figure shows %CV values for n=29 de-identified plasma samples ran in duplicates. %CV's below 15% were marked as green dots; %CV's above 15% and below 20% were marked as yellow dots and for duplicates that had %CV over 20% dots were marked in red colour. Dotted line represents 20% CV border. Source data are provided as a Source Data file.

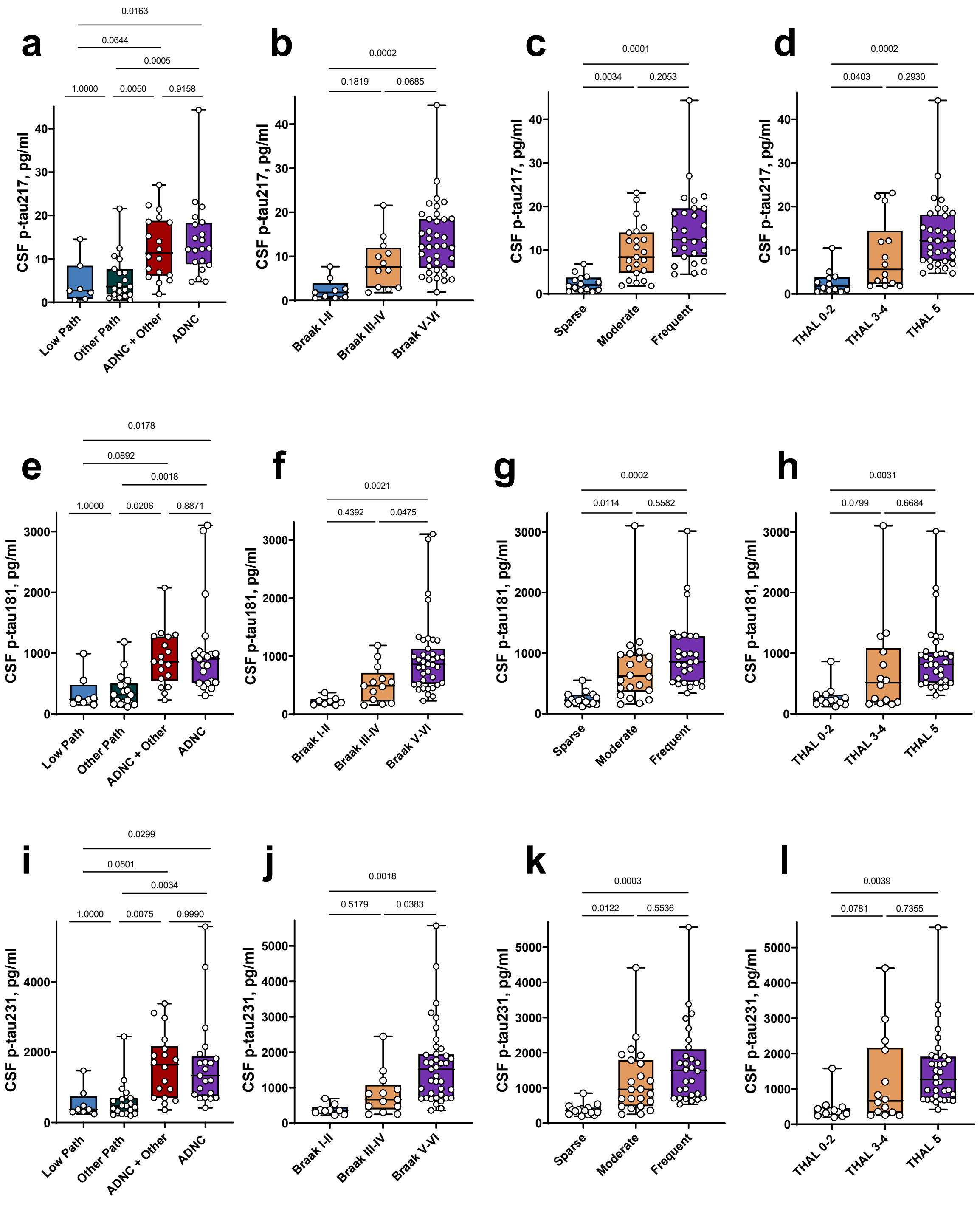

Supplementary Figure 6. Clinical performance of CSF p-tau217, p-tau181 and p-tau231 in the UCSD-Neuropathology cohort.
The figure shows CSF biomarkers levels according to diagnostic groups, amyloid pathology, and Braak staging of neurofibrillary tangles **(a), (e), (i)** CSF biomarker levels according to Alzheimer's disease neuropathologic change (ADNC) categorization. The groups include those with low ADNC pathology - Low Path (non ADNC), Other Path ADNC and ADNC with concomitant neurodegenerative pathologies (ADNC + Other). **(b), (f), (j)** CSF biomarkers levels separated based on Braak staging characterization given at autopsy - Braak I-II, Braak III-IV, Braak V-VI. **(c), (g), (k)** CSF biomarkers concentrations according to the CERAD scores

of Aβ plaques – Sparse, Moderate, Frequent. **(d), (h), (l)** CSF biomarkers concentration across different Thal phases - THAL 0-2, THAL 3-4, THAL 5. Box plots are shown as median and interquartile range (IQR), boundaries of the whiskers are minimum and maximum values. Analysis of variance (ANOVA) with Tukey's post-hoc test was used to compare differences between groups, after adjusting for sex, age, and CSF/plasma collection-to-death intervals. Pairwise comparisons were adjusted for multiple comparisons. Source data are provided as a Source Data file.
