# Supplementary material for: Plasma p-tau212 antemortem diagnostic performance and prediction of autopsy verification of Alzheimer’s disease neuropathology
Source: Nat Commun. 2024 Mar 23;15:2615. doi: 10.1038/s41467-024-46876-7 (PMC10960791; doi:10.1038/s41467-024-46876-7)
Supplement: Supplementary file 3 — Reporting Summary [file 41467_2024_46876_MOESM3_ESM.pdf]

nature portfolio

Corresponding author(s): Przemysław R. Kac

Last updated by author(s): Feb 21, 2024

Reporting Summary

Nature Portfolio wishes to improve the reproducibility of the work that we publish. This form provides structure for consistency and transparency in reporting. For further information on Nature Portfolio policies, see our Editorial Policies and the Editorial Policy Checklist.

Statistics

For all statistical analyses, confirm that the following items are present in the figure legend, table legend, main text, or Methods section.

n/a	Confirmed	
☐	☒	The exact sample size (*n*) for each experimental group/condition, given as a discrete number and unit of measurement
☐	☒	A statement on whether measurements were taken from distinct samples or whether the same sample was measured repeatedly
☐	☒	The statistical test(s) used AND whether they are one- or two-sided<br>*Only common tests should be described solely by name; describe more complex techniques in the Methods section.*
☐	☒	A description of all covariates tested
☐	☒	A description of any assumptions or corrections, such as tests of normality and adjustment for multiple comparisons
☐	☒	A full description of the statistical parameters including central tendency (e.g. means) or other basic estimates (e.g. regression coefficient) AND variation (e.g. standard deviation) or associated estimates of uncertainty (e.g. confidence intervals)
☐	☒	For null hypothesis testing, the test statistic (e.g. *F*, *t*, *r*) with confidence intervals, effect sizes, degrees of freedom and *P* value noted<br>*Give P values as exact values whenever suitable.*
☒	☐	For Bayesian analysis, information on the choice of priors and Markov chain Monte Carlo settings
☒	☐	For hierarchical and complex designs, identification of the appropriate level for tests and full reporting of outcomes
☐	☒	Estimates of effect sizes (e.g. Cohen's *d*, Pearson's *r*), indicating how they were calculated

Our web collection on statistics for biologists contains articles on many of the points above.

Software and code

Policy information about availability of computer code

Data collection	Thermo Scientific Xcalibur 4.5 and Tune 3.5 Software was used.
Data analysis	Statistical analyses were performed with Prism version 9.5 and R Studio version 2023.3.0.386 (packages pROC, PMCMRplus and emmeans - code available at https://zenodo.org/records/10657935). Microscope images were analysed using FIJI ImageJ software version 2.13.1. LC-MS/MS acquisitions were processed using Mascot Daemon v2.6/Mascot Distiller v2.6.3 (both Matrix Science) for charge and isotope deconvolution before submitting searches using Mascot search engine v2.6.1. Quantitative analysis was performed using Skyline v22.2.0.257 (MacCoss Lab).

For manuscripts utilizing custom algorithms or software that are central to the research but not yet described in published literature, software must be made available to editors and reviewers. We strongly encourage code deposition in a community repository (e.g. GitHub). See the Nature Portfolio guidelines for submitting code & software for further information.

Data

Policy information about availability of data

All manuscripts must include a data availability statement. This statement should provide the following information, where applicable:

- Accession codes, unique identifiers, or web links for publicly available datasets
- A description of any restrictions on data availability
- For clinical datasets or third party data, please ensure that the statement adheres to our policy

Custom database used for peptide search was cointaing only human tau isoforms from Uniprot (Uniprot ID: P10636). Additional data is provided in the supplementary data. Source data are provided with this file. Any other blinded anonymized data is available on reasonable request from the corresponding author. Request will be reviewed by the investigators and respective institutions to verify if data transfer is in the agreement with EU or USA legislation on the general data protection or is subject to any intellectual property or confidentiality obligations.

Research involving human participants, their data, or biological material

Policy information about studies with human participants or human data. See also policy information about sex, gender (identity/presentation), and sexual orientation and race, ethnicity and racism.

Reporting on sex and gender	We used term "sex" in the manuscript, basing on self-reporting. In post-mortem cohorts "sex" was used as a covariate in statistical analysis. In five analyzed cohorts, samples were coming both from man (n=173) and woman (n=215), therefore we believe that findings presented here apply to both sexes.
Reporting on race, ethnicity, or other socially relevant groupings	No relevant socially constructed variables or categorizations variables were used in the manuscript.
Population characteristics	Detailed cohort characteristics are presented in Table 1 and Extended Data Tables 4-7. To summarize, studies included a total of n=388 participants from five independent cohorts. The Baltimore Longitudinal Study of Aging (BLSA)-Neuropathology cohort (n=47; Extended Table 6) classified participants as autopsy-verified-AD (n=20), asymptomatic-AD (ASYMAD) (n=15) and non-AD controls (n=12). The University of California San Diego (UCSD)-Neuropathology cohort (n=67; Extended Data Table 7) included autopsy-confirmed high-ADNC (n=21), low ADNC (n=8), other neuropathologies (n=19) and mixed high-ADNC with other neuropathologies (n=19). The Gotheburg (n=30; Extended Data Table 4) and the Polish (n=95; Extended Data Table 5) cohorts consisted of individuals with abnormal CSF core biomarker profiles for AD and biomarker-negative controls. The Slovenian memory clinic cohort from the University Medical Center, Ljubljana, (n=149; Table 1) included patients in the AD continuum, that is, MCI due to AD (n=41), and AD dementia (n=62), as well as participants with subjective cognitive decline (n=24) and MCI not due to AD (n=22).
Recruitment	Specific recruitment criteria are given in cohort descriptions in methods section.
Ethics oversight	This study was performed according to the Declaration of Helsinki. The Gothenburg Discovery cohort that used de-identified leftover clinical samples was approved by the ethics committees at the University of Gothenburg (#EPN140811). Studies including the Polish cohort were approved by the local ethical committee (KB-380/2017) at the University of Wroclaw. The UCSD-Neuropathology cohort was reviewed and approved by the human subject review board at UCSD (IRB 170957). Informed consent was obtained from all patients or their caregivers consistent with California State law. The study in Slovenian cohort was approved by the Medical Ethics Committee of the Republic of Slovenia, Ministry of Health (0120-342/2021/6). The BLSA studies have ongoing approval from the Institutional Review Board of the National Institute of Environmental Health Science, National Institute of Health (IRB 03AG0325). Anatomical Gift Act for organ donation and a repository consent was signed by the participants to allow sharing the data and biospecimens.

Note that full information on the approval of the study protocol must also be provided in the manuscript.

Field-specific reporting

Please select the one below that is the best fit for your research. If you are not sure, read the appropriate sections before making your selection.

☒ Life sciences ☐ Behavioural & social sciences ☐ Ecological, evolutionary & environmental sciences

For a reference copy of the document with all sections, see nature.com/documents/nr-reporting-summary-flat.pdf

Life sciences study design

All studies must disclose on these points even when the disclosure is negative.

Sample size	In n=388 participants from five different cohorts, we analyzed all available plasma/CSF samples. Sample number was not predetermined before, and was based on the availability of fluid volumes. The results are strongly positive and convergent across the cohorts, indicating that we had sufficient number of samples.
Data exclusions	In Polish cohort, we included only participants who have available measurements for both plasma and CSF, to provide direct comparison

Data exclusions	between matrices. In supplementary Data Figure 5. we excluded n=1 sample which had no available replicate due to technical error.
Replication	Utility of p-tau212 as a biomarker for Alzheimer's Disease was analysed in five independent cohorts, including multiple comparisons with biomarkers that already have established efficacy. Results were replicated in all the cohorts.
Randomization	This is an observational study and no allocation into experimental groups were performed, therefore randomization is not relevant to this study.
Blinding	All biomarker analyses were performed by the researchers that were blinded to the clinical data of the participant.

Reporting for specific materials, systems and methods

We require information from authors about some types of materials, experimental systems and methods used in many studies. Here, indicate whether each material, system or method listed is relevant to your study. If you are not sure if a list item applies to your research, read the appropriate section before selecting a response.

Materials & experimental systems

n/a	Involved in the study	
☐	☒	Antibodies
☒	☐	Eukaryotic cell lines
☒	☐	Palaeontology and archaeology
☒	☐	Animals and other organisms
☒	☐	Clinical data
☒	☐	Dual use research of concern
☒	☐	Plants

Methods

n/a	Involved in the study	
☒	☐	ChIP-seq
☒	☐	Flow cytometry
☒	☐	MRI-based neuroimaging

Antibodies

Antibodies used

For p-tau212 and p-tau217 antibody generation, custom-designed p-tau peptides phosphorylated at threonine-212 and threonine-217 with C-terminal tetanus toxin sequences were synthesized (Severn Biotech, UK). These peptides were used for the immunization of sheep and the monoclonal antibody generation process followed as described (Osborne et al., Hybridoma, 1999). Afterwards, candidate hybridomas were selected based on binding to specifically phosphorylated peptides, as described in Figure 1. Antibody design, generation and validation were performed at Bioventix Plc (Surrey, United Kingdom).

For p-tau181 and p-tau231 antibody generation used for direct ELISA, custom-designed p-tau peptides phosphorylated at threonine-181 and threonine-231 with C-terminal tetanus toxin sequences were synthesized (GenScript, UK). These peptides were used for the immunization of sheep and the monoclonal antibody generation process followed as described (Osborne et al., Hybridoma, 1999). Afterwards, candidate hybridomas were selected based on binding to specifically phosphorylated peptides, as described in Figure 1. Antibody design, generation and validation were performed at Bioventix Plc (Surrey, United Kingdom).

For detection in Simoa assays, a biotynylated mouse monoclonal antibody raised against the N-terminal region of tau (Tau12; BioLegend, #SIG-39416) was used.

For p-tau181 Simoa assay, anti-p-tau181 Tau antibody (Thermo Fisher, catalog number: MN1050) was used.

Anti-p-tau217 (Thermo Fisher; catalog number 44-744) was used for tissue staining and as a capture for p-tau217 CSF Simoa assay.

For p-tau231 Simoa assay, monoclonal mouse antibodies were generated using a synthetic peptide (K224KVAVVR(pT)PPKSPSSAK240C) as a KLH-coupled antigen, numbered according to full-length tau-441 phosphorylated on threonine-231. Candidate hybridomas were selected on brain extracts of AD and control brain tissue. The final cloned and purified monoclonal antibody, ADx253, was characterized on synthetic peptides spanning amino acids threonine 217 till serine 241 of full-length tau for its affinity, its phospho-specificity using both phosphorylated and non-phosphorylated peptides and its preferred selectivity in which position 232 was replaced by a Pip, to simulate cis-selectivity of ADx253.

AT8 antibody against epitope p-tau202/p-tau205 (Thermo Fisher Scientific; catalog number MN1020) was used for tissue staining.

Secondary antibodies for tissue staining were obtained from Thermo Fisher Scientific (anti-sheep Alexa-fluor 488; A-11015; anti-rabbit Alexa-fluor 647; catalog number A-21245 and anti-mouse Alexa-fluor 647; catalog number A-21235)

For the plasma IP-MS Tau12, HT7 (Tau Monoclonal Antibody, MN1000, Thermofisher), BT2 (Tau Monoclonal Antibody MN1010, Thermofisher were used)

Validation

P-tau181, p-tau231 and commercially available p-tau217 antibodies were previously validated (Karikari TK et al., Lancet Neurol 2020; Ashton NJ et. al Acta Neuropathol, 2021; Karikari TK et al., Alzheimers dement, 2021 respectively)
Validation of p-tau212 assay is presented in the article. The plasma p-tau217 assay validation is detailed in (González-Ortiz et al. Alzheimers dement,2023).
Staining protocol validation was previously described (Koutarapu et al., Brain Communications, 2022)

Mass spectrometric analysis of plasma samples was performed as previously described (Montoliu-Gaya et al., Nat. Aging, 2023)

Mass spectrometric analysis of phosphorylated tau peptides was previously validated in article (Cicognola et al. Acta Neuropathol; 2019)
Tau12 (https://www.biolegend.com/fr-ch/products/purified-anti-tau-6-18-antibody-11569)
AT8 (https://www.thermofisher.com/antibody/product/Phospho-Tau-Ser202-Thr205-Antibody-clone-AT8-Monoclonal/MN1020)
BT2 (https://www.thermofisher.com/antibody/product/Tau-Antibody-clone-BT2-Monoclonal/MN1010)
HT7 (https://www.thermofisher.com/antibody/product/Tau-Antibody-clone-HT7-Monoclonal/MN1000)
anti-sheep Alexa-fluor 488 https://www.thermofisher.com/antibody/product/Donkey-anti-Sheep-IgG-H-L-Cross-Adsorbed-Secondary-Antibody-Polyclonal/A-11015
anti-rabbit Alexa-fluor 647 https://www.thermofisher.com/antibody/product/Goat-anti-Rabbit-IgG-H-L-Highly-Cross-Adsorbed-Secondary-Antibody-Polyclonal/A-21245
anti-mouse Alexa-fluor 647 https://www.thermofisher.com/antibody/product/Goat-anti-Mouse-IgG-H-L-Cross-Adsorbed-Secondary-Antibody-Polyclonal/A-21235

Plants

Seed stocks

Report on the source of all seed stocks or other plant material used. If applicable, state the seed stock centre and catalogue number. If plant specimens were collected from the field, describe the collection location, date and sampling procedures.

Novel plant genotypes

Describe the methods by which all novel plant genotypes were produced. This includes those generated by transgenic approaches, gene editing, chemical/radiation-based mutagenesis and hybridization. For transgenic lines, describe the transformation method, the number of independent lines analyzed and the generation upon which experiments were performed. For gene-edited lines, describe the editor used, the endogenous sequence targeted for editing, the targeting guide RNA sequence (if applicable) and how the editor was applied.

Authentication

Describe any authentication procedures for each seed stock used or novel genotype generated. Describe any experiments used to assess the effect of a mutation and, where applicable, how potential secondary effects (e.g. second site T-DNA insertions, mosiacism, off-target gene editing) were examined.
